# Machine Learning Identifies a Parsimonious Differential Equation for Myricetin Degradation from Scarce Data

**DOI:** 10.3390/foods14122135

**Published:** 2025-06-18

**Authors:** Andrew Fulkerson, Ipek Bayram, Eric A. Decker, Carlos Parra-Escudero, Jiakai Lu, Carlos M. Corvalan

**Affiliations:** 1 Transport Phenomena Laboratory, Department of Food Science, Purdue University, West Lafayette, IN 47906, USA; 2 Department of Food Science, University of Massachusetts, Amherst, MA 01003, USAcparraescude@umass.edu (C.P.-E.)

**Keywords:** machine learning, neural differential equations, sparse symbolic regression, food chemistry, antioxidant degradation, shelf life prediction

## Abstract

Accurately modeling the degradation of food antioxidants in oils is essential for understanding oxidative stability and improving food shelf life. This study presents an innovative machine learning approach integrating neural differential equations and sparse symbolic regression to derive a parsimonious differential equation for myricetin degradation in stripped soybean oil. Despite being trained on a small experimental dataset, the model successfully predicts degradation trends across a wide range of initial concentrations and extrapolates beyond the learning data. This capability demonstrates the robustness of machine learning for uncovering governing equations in complex food systems, particularly when experimental data is scarce. Our findings provide a framework for improving antioxidant efficiency in food formulations.

## 1. Introduction

Food scientists need to characterize the breakdown of antioxidants in food oils to understand and control factors affecting lipid oxidation and stability, and thus the shelf life of foods. Mathematical modeling provides a critical foundation for quantitative analysis and has long been used to study quality changes in food, including oxidative stability in emulsions [1,2,3,4,5,6]. Traditional modeling approaches often rely on fitting experimental observations to pre-assumed models based on expert knowledge [1]. While this approach has successfully forecasted food shelf life, it can be challenging when experimental data is scarce [7].

Myricetin, a flavonol with potent antioxidant properties, plays a key role in enhancing the oxidative stability of bulk oil [8] and food emulsions [9,10]. Its ability to inhibit lipid oxidation, particularly of linoleic acid—one of the major components of soybean oils—makes it highly relevant in food preservation studies [11,12]. The inhibition of lipid oxidation prevents the formation of free radicals, which can lead to emulsion breakdown [12,13,14].

To address the challenge of extracting governing equations from limited experimental observations, we introduce an innovative machine learning approach integrating neural differential equations (NDEs) and sparse symbolic regression. NDEs learn a neural network representation of the degradation rate, as they are a powerful new class of models that integrate dynamical systems and deep learning [15,16,17]. They have been successfully applied to problems such as reduced-order models of turbulence [18] and optimizing treatment strategies for HIV [19]. By augmenting limited experimental data with NDEs, we enable sparse symbolic regression [20] to identify a governing equation for the degradation process without prior assumptions about the underlying dynamics. Sparse symbolic regression, which focuses on deriving parsimonious differential equations [21], has been successfully applied to diverse scientific domains ranging from epidemiology [22] to complex fluid dynamics [23].

In this study, we leverage machine learning to develop a predictive differential model for myricetin degradation in soybean oil using a small experimental dataset. Our approach seamlessly integrates neural differential equations and sparse symbolic regression to extract a governing equation that accurately describes myricetin degradation across various initial concentrations. The results demonstrate that this model extrapolates beyond the training data, offering a powerful tool for uncovering reduced-order equations in food systems where experimental data is scarce.

## 2. Material and Methods

Soybean oil was obtained from a store in Hadley, MA, USA, and medium chain triacylglycerol (MCT) was purchased from Warner Graham Company (Baltimore, MD, USA), and was kept frozen at −80 °C until use. Hexane was bought from Fisher Scientific (Fair Lawn, NJ, USA), and myricetin was purchased from Combi-Blocks Inc. (San Diego, CA, USA). Activated charcoal, silicic acid, ethanol, and 1,4-dioxane were purchased from Sigma-Aldrich (St. Louis, MO, USA).

### 2.1. Preparation of Stripped Soybean Oil

The isolation of soybean oil triacylglycerols was performed using the method described by Boon et al. [24] to eliminate the impact of endogenous antioxidants and prooxidants. Prior to the experiment, silicic acid was washed seven to eight times to remove any impurities and dried in an incubator at 125 °C for 24 h. The column was packed with 22.5 g of silicic acid, 5.25 g of activated charcoal, and 22.5 g of silicic acid, with each layer being dissolved in approximately 40 mL of hexane, followed by the elution of excess hexane. Soybean oil (30 g) and hexane (30 mL) were combined and added into the packed chromatographic column. Stripped soybean oil (SSO) was collected by eluting a total of 270 mL of hexane over the course of three to four hours. A round-bottom flask used to collect SSO plus hexane mixture was placed on ice to prevent further lipid oxidation during the stripping process. Similarly, aluminum foil was used to cover the chromatographic column to minimize light-induced oxidation. At a temperature of 27 °C, hexane was separated from SSO utilizing a vacuum rotary evaporator (Model RE 111, Buchi, Flawil, Switzerland). The recovered SSO was flushed with nitrogen for 10 min to remove any traces of hexane, and then stored at −80 °C until use.

### 2.2. Preparation of Samples for Oxidation Study

The lipid oxidation study used a 50:50 mixture of oxidizable stripped soybean oil (SSO) and non-oxidizable medium chain triacylglycerols (MCT) to reduce waste associated with excess oil stripping. Ethanol stock solution of myricetin was prepared and pipetted into the oil blend to achieve final myricetin concentrations of 10, 25, 50, 100, and 250 μM. The samples were mixed on a magnetic stirrer at 300 rpm at 4 °C overnight to ensure that the myricetin was distributed evenly throughout the oil. The samples (0.5 mL) were placed in 10 mL Supelco GC headspace vials, capped with aluminum caps with PTFE/silicone septa, and stored in the dark at 60 °C. Three vials from each sample group were taken on a regular basis to analyze the myricetin degradation kinetics.

### 2.3. Measurement of Myricetin Concentrations

The concentration of myricetin during oxidation was determined periodically using a Shimadzu Prominence-i LC-2030C HPLC (Shimadzu, Kyoto, Japan) having a Supelcosil LC-Diol column (25 cm by 4.0 mm, particle size 5 μM, Bellefonte, PA, USA), a Supelguard LC-Diol guard column (2 cm by 4.0 mm, particle size 5 μM) and an in-built photodiode array detector (PDA). The samples were prepared by dissolving 125 μL oil samples in 1 mL of 1,4-dioxane and filtering them into the HPLC vials using 0.22 μM PTFE syringe filters (Fisher Scientific, Pittsburgh, PA, USA). A total of 40 μL of samples were injected with an autosampler into the analytical column at 37 °C. The isocratic flow of 40% hexane and 60% 1,4-dioxane at a mobile phase flow rate of 1 mL/min was used to detect myricetin. Myricetin was identified and quantified at about 3.9 min of retention time over a 10 min separation period at 370 nm, the wavelength of maximal absorbance. The remaining myricetin percentage on a given day was calculated by dividing the measured peak area by the measured peak area of day zero. Sampling time points were selected based on prior studies of antioxidant degradation kinetics in bulk oils, which typically occur over several days [10,25]. This schedule ensured adequate resolution of the degradation profile while minimizing unnecessary sampling.

### 2.4. Machine Learning Approach for Myricetin Degradation

To derive a governing equation for myricetin degradation, we employed a machine learning framework combining neural differential equations (NDEs) and sparse symbolic regression. The NDE establishes a data-driven representation of the degradation process, allowing us to model the rate of change using a neural network. Sparse regression then extracts a parsimonious differential equation by selecting relevant monomial terms through regularization techniques. A detailed description of this approach, including implementation specifics, is provided in Section 3.1 and Section 3.2 of Section 3.

## 3. Results and Discussion

To demonstrate our machine learning approach, we first measured the degradation of myricetin in soybean oil over a range of initial concentrations $c_{0}$ from 10 to 100 μM, as described in Section 2, Methods. Table A1 in Appendix A shows the measurement results. The concentration data y(t) were normalized by dividing each data point by the corresponding initial concentration.

**Figure 1:**
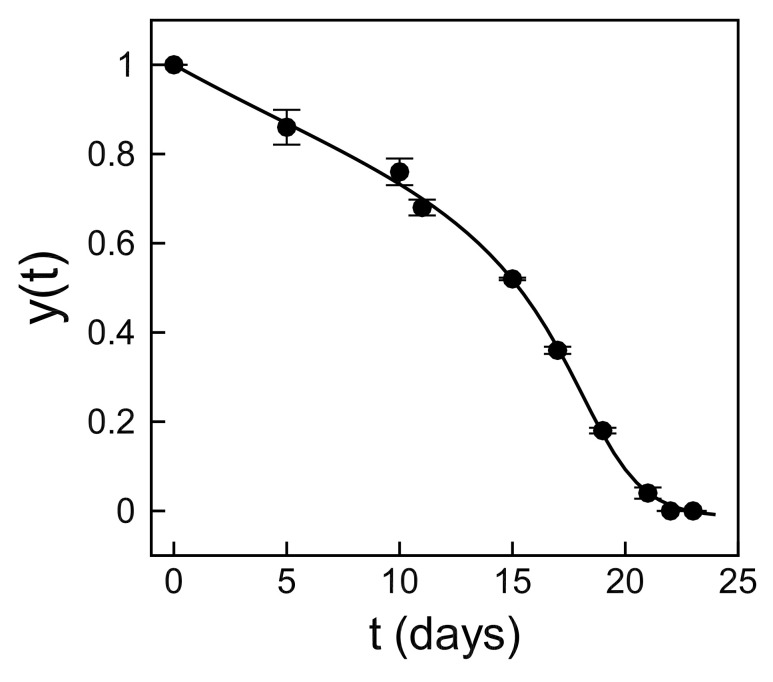
Solution of the neural differential equation (NDE). The NDE (solid line) closely matches the degradation dynamics observed in a small experimental dataset (symbols) for an initial myricetin concentration of 50 μ M.

As discussed in the Introduction, our approach to learning the myricetin dynamics consists of two steps:

- First, we solve a *neural differential equation* (NDE) [15,16] to augment the limited experimental observations. We demonstrate the effectiveness of this method using the observations for the intermediate myricetin concentration of 50 μM (Figure 1).
- Then, we use the NDE solution in a *sparse regression problem* [21] to discover a parsimonious differential equation underlying the degradation dynamics (Equation (3)).

We elaborate on the results of the neural differential equation, the sparse regression, and the model’s predictions in the following sections. For completeness, we also outline a conventional approach based on assuming a model beforehand in Appendix B.

### 3.1. Neural Differential Equation

Standard machine learning models require a large amount of training data. This can be a limitation when only scarce experimental data is available. To overcome this limitation, we first built a data-driven representation of the myricetin dynamics using a neural differential equation to augment the experimental dataset. An NDE is essentially a differential equation in which the derivative is specified by a neural network [16]. The extra information added by imposing a differential form allows the neural network to learn from a smaller amount of training data.

The NDE used in this work is a simple ordinary differential equation for the normalized myricetin concentration y(t), where the degradation rate is described by a neural network $f_{\theta }$ as

(1) $$\frac{dy}{dt}\left(t\right)=f_{\theta }\left(y\left(t\right)\right),\;y\left(0\right)=1.$$

The subscript θ represents the vector of parameters (weights and biases) of the neural network. We trained Equation (1) on experimental measurements to learn the parameters θ that represent the underlying dynamics. The NDE (Equation (1)) was solved using a hybrid numerical approach. The dynamics of ordinary differential equations were integrated through an adaptive Runge–Kutta 4th/5th order (RK45) scheme, while the unknown term $f_{\theta }$ was parameterized by a neural network. Specifically, we used a fully connected feedforward neural network that features a shallow architecture: an input layer, two hidden layers, each with five neurons, and an output layer. The hidden layers used the Rectified Linear Unit (ReLU) activation function, while the output layer, yielding a single output for each time step, employed a linear activation suitable for the final regression. This parsimonious architecture was chosen following an empirical evaluation of networks with varying depths up to 50 layers and widths from 3 to 100 neurons, balancing model capacity and generalization. The network parameters were optimized through backpropagation using automatic differentiation [26,27] to minimize the loss of the mean squared error (MSE) between the numerical solution and the experimental measurements (at $c_{0}=50$ μM). Optimization was performed using the Adam optimizer [28] with an initial learning rate of 0.001. Due to the limited size of the dataset, the full batch gradient descent was utilized, achieving convergence in fewer than 500 iterations with a final training MSE of $1.2\times 10^{-5}$. The models were implemented in Python 3.12 using Keras [29] with the TensorFlow 2.14 backend [30] and trained on a Nvidia GeForce RTX GPU, NVIDIA Corp., Santa Clara, CA, USA.

Figure 1 illustrates the model-agnostic solution of the neural differential equation trained on measurements for the intermediate initial concentration of 50 μM. The solid line shows the learned degradation dynamics, while the symbols show the experimental measurements. The NDE was able to learn a good representation of the dynamics, even though it was trained on a small experimental dataset using a shallow network. Importantly, this is a model-agnostic representation, which avoids imposing an arbitrary analytical form to describe the degradation rate in the differential equation. The neural network $f_{\theta }$ cannot generalize nor predict beyond the learning data, but it does provide an augmented high-dimensional representation of the experimental data and their derivatives. These high-dimensional solutions can be used to find an analytical expression for the differential equation using sparse regression.

### 3.2. Sparse Identification of Nonlinear Dynamics

In a second step, we used sparse regression to identify an analytical expression for the differential degradation model able to generalize to different initial concentrations. We applied the Sparse Identification of Nonlinear Dynamics (SINDy) algorithm. SINDy is an effective machine-learning technique for extracting parsimonious dynamics from high-dimensional time-series data. A comprehensive description of the SINDy algorithm, along with its source code implementation, is available in the original publication by Champion et al. [31]. Accordingly, this section provides a brief outline within the context of our dataset.

The implementation is straightforward using the information provided by Equation (1). We construct two high-dimensional input vectors y and $\dot{y}$. The vector y is the augmented vector of concentrations $y=\left[\begin{array}{c} y\left(t_{1}\right)\;y\left(t_{2}\right)\;\ldots \;y\left(t_{m}\right) \end{array}\right],$ generated by sampling the NDE solution y(t) at several time steps $t_{1},\ldots ,t_{m}$ with m≫1. The vector $\dot{y}$ is the corresponding vector of time derivatives $\dot{y}=\left[\begin{array}{c} f_{\theta }\left(y\left(t_{1}\right)\right)\;f_{\theta }\left(y\left(t_{2}\right)\right)\;\ldots \;f_{\theta }\left(y\left(t_{m}\right)\right) \end{array}\right],$ generated by feeding the vector y to the trained neural network $f_{\theta }\left(y\right)$. Note that $f_{\theta }$ represents the degradation rate (i.e., the derivative) in the NDE.

The SINDy algorithm then searches for a linear combination of basis functions of y that best approximates the vector $\dot{y}$. The basis functions are typically polynomials or trigonometric functions if oscillatory behavior is expected. In this work, we are interested in a mass-action type solution, so we use basis functions in the vector space A(y) of monomials of y. In other words, each term in the array A(y) represents a potential mass-action order for the degradation kinetics. The SINDy algorithm then solves the regression problem

(2) $$\dot{y}=A\left(y\right)\cdot b$$

using sparsity-promoting regularization, which penalizes the number of active basis functions [31]. Solution b is a sparse vector of coefficients that determine which terms are active in the array of polynomials A(y). One way to promote sparsity in the regression problem is to use $\ell_{0}$-norm regularization, which penalizes the number of nonzero entries in the array. However, $\ell_{0}$ regularization is nonconvex, making it difficult to identify the global optimum of the regression problem. A better choice is the $\ell_{1}$ penalty, known as the least absolute shrinkage and selection operator (LASSO) algorithm, which is the convex approximation to the $\ell_{0}$ penalty [32]. LASSO can be computationally intensive, so we implemented the more computationally efficient sequentially thresholded least-squares (STSL) algorithm [33,34], as used in the standard SINDy algorithm [31]. Another common form of computationally efficient regularization is ridge regression, which uses an $\ell_{2}$ penalty. However, ridge regression does not necessarily promote sparsity, because the $\ell_{2}$ norm does not penalize the number of nonzero coefficients [35].

When applied to the NDE solution for $c_{0}=50$ μM, illustrated in Figure 1, the SINDy algorithm identified three active monomials *y*, $y^{2}$, and $y^{3}$ to represent the degradation rate. Optimizing the coefficients of the three monomials resulted in the following analytical expression for the differential equation:

(3) $$\frac{dy}{dt}=-r\left(y\left(1-Ky\right)+y^{3}\right).$$

This equation describes a cubic correction to a logistic decay of myricetin, with *r* and *K* being the rate coefficient and capacity constant, respectively. The logistic term y(1−Ky) reflects the autocatalytic behavior commonly observed in antioxidant degradation, while the cubic term $y^{3}$ captures the dynamics of late-stage decay [36,37]. These components align with known chemical mechanisms in lipid oxidation systems. The values of *r* and *K* characterized for the four initial concentrations (Table A1) are summarized in Table 1.

### 3.3. Model Predictions

**Figure 2:**
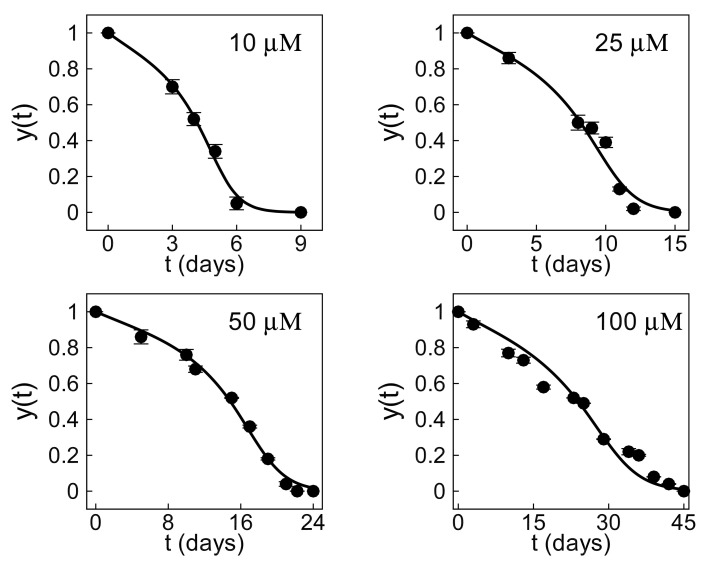
Myricetin degradation over a range of initial concentrations from 10 to 100 μM. The solutions of the differential model represented by Equation (3) (solid line) are in good agreement with the experimental data (symbols).

The rate coefficients in Table 1 decrease rapidly with the initial concentration. This means that shelf life increases as the initial concentration $c_{0}$ of the antioxidant increases. The capacity constant, on the other hand, is largely independent of the initial concentration. This indicates that the degradation is self-similar in normalized concentration, although not in time, which scales with the rate coefficient. Figure 2 compares the predictions of the differential model (solid line) to the experimental data (symbols) for initial concentrations of 10 to 100 μM, spanning a shelf life of approximately one to six weeks. The differential equation with just two parameters can describe the degradation process over a decade-long range of initial concentrations. This suggests that the uncovered equation is a robust model of the degradation process.

**Figure 3:**
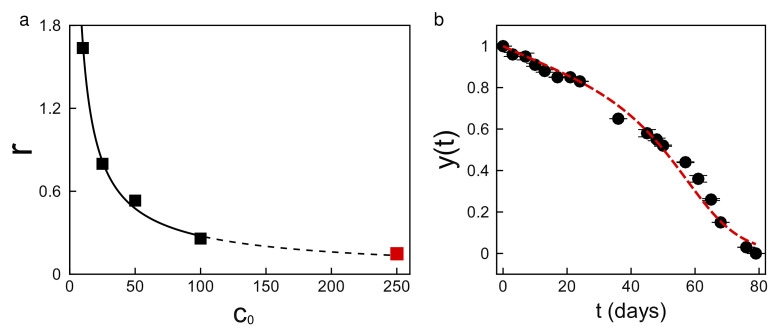
The differential model makes shelf-life predictions well beyond the available data. (**a**) The continuous line shows a power-law fit of the rate coefficients in Table 1. The dashed line shows the extrapolation of the fit to 250 μM. (**b**) The differential model predicts the degradation of the antioxidant for a shelf-life of more than two and a half months using the extrapolated parameter.

We further tested Equation (3) by extrapolating its predictions to a sample with an initial concentration much larger than those in Table 1. First, we established that the rate coefficient *r* for the four concentrations in Table 1 decreases approximately as a power-law $r=10.1\;c_{0}^{-0.781}$, as shown by the solid line of Figure 3a. This allowed us to directly extrapolate the rate coefficient to more concentrated samples, as represented by the dashed line in Figure 3a. In a demanding test, we used the rate coefficient extrapolated to $c_{0}=250$ μM (red symbol), which is 2.5-times larger than the highest initial concentration in the training set. The result of the test is shown in Figure 3b, where the dashed line represents the model predictions and the solid symbols represent the experimental data. The figure shows that the model is able to estimate the degradation of the antioxidant over time up to a shelf-life of more than two and a half months.

Food antioxidants degrade through a complex process involving many finely coordinated chemical reactions [38,39,40]. A complete understanding of the process would require identifying all intermediate species involved and characterizing a large number of rate constants, which is often impractical. Instead, Equation (3) is a reduced-order model with only two kinetic parameters that can be characterized from our observations. This parsimonious model can describe and predict myricetin degradation over a large range of concentrations, as demonstrated in Figure 2 and Figure 3. However, it does not provide a complete mechanistic understanding of the full kinetics. Despite this limitation, the reduced-order model does more than make meaningful quantitative predictions. The model also uncovers important qualitative features of the underlying dynamics, which are not immediately apparent from the experimental data.

First, the model identified three distinct stages of degradation: initiation, propagation, and termination (Figure 2 and Figure 3b). Most food antioxidants show this pattern [25,41,42,43]. In our case, initiation is a brief early stage, accounting for about 6% of the reaction’s progress. At this point, the normalized concentration decreases to y(t)≈0.94, which corresponds to the first inflexion point $1/3(K+\sqrt{K^{2}-3})$ of Equation (3). The reaction rate decreases with time during this brief initial stage. Propagation is a longer stage, occurring at intermediate times, until the concentration decreases to y(t)≈0.36. This value corresponds to the second inflexion point $1/3(K-\sqrt{K^{2}-3})$ of Equation (3). The reaction rate increases with time during this stage. Termination is the final stage, proceeding until all of the antioxidant has been consumed. The reaction rate decreases with time during this last stage.

Second, the model identified a subtle mechanism: antioxidants often decay at later times through an autocatalytic process [37,44]. This is consistent with the characteristic sigmoidal decay observed at later times in Figure 2 and Figure 3b. More precisely, as the myricetin concentration decreases with time, the contribution of the cubic nonlinearity in Equation (3) becomes progressively smaller. Eventually, the degradation is governed by the logistic decay $dy/dt=-r\left(y\left(1-Ky\right)\right)$, which is characteristic of autocatalytic reactions [36,45,46].

The model’s ability to learn these features directly from data provides insights into the underlying dynamics of the myricetin degradation process.

## 4. Conclusions

Understanding how antioxidants degrade in oil and oil emulsions is essential for extending the shelf life of many foods. In this study, we introduce a machine learning approach for discovering differential equations that model antioxidant breakdown without relying on prior assumptions about the underlying dynamics. We demonstrate this method by identifying a parsimonious, two-parameter differential equation that accurately describes the degradation of myricetin in stripped soybean oil. Despite being trained on limited experimental data, the model captures degradation behavior across a wide range of initial concentrations (10–100 μM) and generalizes well beyond the training set, up to 250 μM. Although data-driven, the model’s structure reflects known chemical mechanisms, including autocatalytic behavior and late-stage decay dynamics commonly associated with lipid oxidation. These features improve the interpretability of the model and its relevance to food chemistry. While the current model focuses on myricetin in stripped soybean oil at a fixed temperature, its robustness across varying concentrations suggests broader applicability to other antioxidants, oil systems, and environmental conditions. However, it does not yet account for key environmental variables such as temperature, oxygen partial pressure, or interfacial dynamics, factors known to influence antioxidant degradation. Incorporating these parameters, either through additional data or by integrating mechanistic insights from food chemistry, could significantly improve the model’s predictive power and extend its utility to a wider range of food formulations and storage conditions. Finally, although we applied this approach to antioxidant degradation, it holds strong potential for modeling other complex food processes where traditional methods struggle due to limited data.

## Figures and Tables

**Table 1:** Myricetin degradation rate *r* and capacity constant *K* for initial concentrations $c_{0}=10$, 25, 50, and 100 μM.

Concentration $c_{0}$ (μM)	Rate *r* (${\mathrm{Day}}^{-1}$)	Capacity *K* (-)
10	1.636	1.947
25	0.799	1.943
50	0.533	1.960
100	0.258	1.938

**Figure A1:**
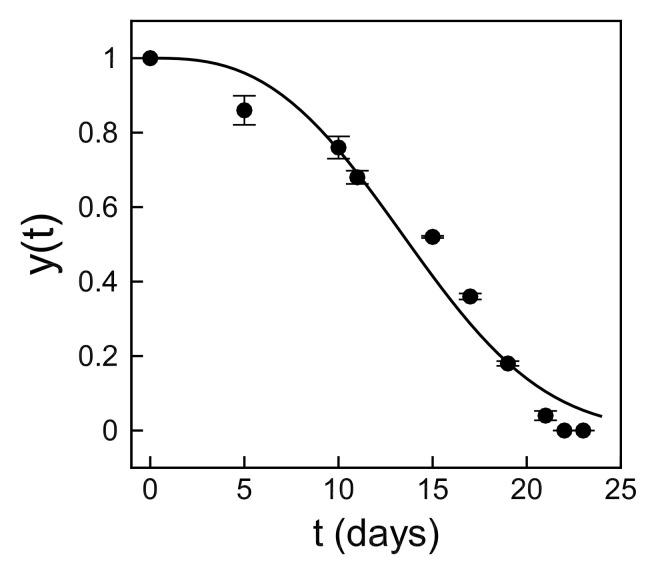
Fitting of the myricetin degradation data (symbols) using the Weibull model (solid line) for an initial myricetin concentration $c_{0}=50$ μM. The fitting parameters are n=7.2 and d=15.7.

**Figure A2:**
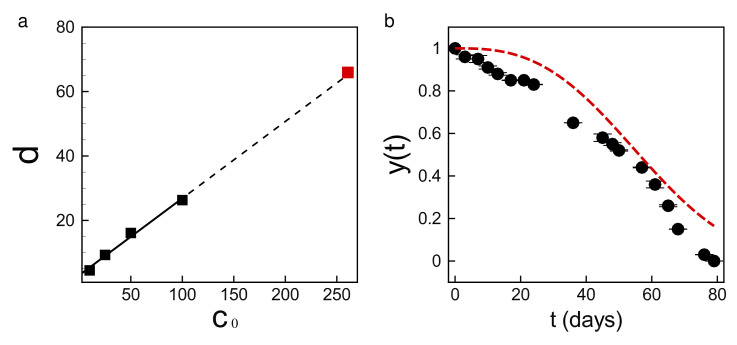
Forecasting myriceting shelf life to $c_{0}=250$ μM using the Weibull model. (**a**) The dashed line shows the extrapolation of the fitting parameter *d* to $c_{0}=250$ μM. The parameter n≈7.2 is essentially constant. (**b**) Shelf life predicted by the Weibull model (dashed line) and corresponding experimental observations (symbols).

## Data Availability

The experimental myricetin degradation datasets are available upon reasonable request.

## Appendix A. Degradation of Myricetin in Soybean Oil at Different Initial Concentrations

The degradation of myricetin in soybean oil was measured over time for a range of initial concentrations $c_{0}$. The results are shown in Table A1. The concentration data was normalized by dividing each data point by the corresponding initial concentration to remove any scale dependence.

**Table A1:** Degradation of myricetin in soybean oil at different initial concentrations. The normalized myricetin concentration y(t) is shown as a function of time for initial concentrations $c_{0}=10$, 25, 50, and 100 μM. The standard deviation was calculated from triplicate measurements.

	Initial Concentration $c_{0}$ (μM)			
Time (Day)	10	25	50	100
3	0.7±0.0396	0.86±0.0314		0.93±0.0189
4	0.52±0.0361			
5	0.34±0.0382		0.86±0.039	
6	0.05±0.0355			
8		0.5±0.0416		
9	0±0	0.47±0.03283		
10		0.39±0.02863	0.76±0.0299	0.77±00.0213
11		0.13±0.01147	0.68±0.0177	
12		0.02±0.0098		
13				0.73±0.0166
15		0±0	0.52±0.0029	
17			0.36±0.008	0.58±0.011
19			0.18±0.0063	
21			0.04±0.0126	
23				0.52±0.0001
24			0±0	0.49±0.0005
25				0.29±0.0017
29				0.22±0.0173
34				0.2±0.0052
36				0.08±0.0004
39				0.04±0.0017
42				0±0

## Appendix B. Fitting Myricetin Degradation Data with the Weibull Model

For completeness, in this section, we exemplify a conventional model-based approach to forecasting the shelf life of myricetin using the degradation data in Table A1. Model-based fitting of experimental observations is a powerful tool with a long history in forecasting food shelf life, but requires choosing a suitable model in advance, often generated from expert knowledge. The discovered differential Equation (3) involves two parameters, so we selected a two-parameter Weibull model (also known as Peleg’s model [47]) for this example. The Weibull model is a commonly used nonlinear model in food science [48,49] and fits the sigmoidal experimental data in Table A1 well (e.g., Figure A1). In previous work [50], we compared several kinetic models—including Weibull, Gompertz, power-law, and logistic—and found that the Weibull model provided the best balance of fitting accuracy (high $R^{2}$, low RMSE), moderate tail behavior, and mathematical simplicity. This made it particularly suitable for extrapolation and interpretation in food degradation studies. The model is described by the non-autonomous differential equation

(A1) $$\frac{dy}{dt}=-by/t^{n},$$

which has the simple analytical solution

(A2) $$y\left(t\right)=\exp\left(-{\left(t/d\right)}^{1-n}\right)$$

where y(t) is the normalized concentration of myricetin, *t* is time, and $d={\left(b/\left(1-n\right)\right)}^{1/(n-1)}$.

Figure A1 shows that the nonlinear Weibull model fits the experimental degradation data well for an initial myricetin concentration of 50 μM, comparable to our results in Figure 1. However, the two figures have fundamentally different interpretations. Fitting in Figure A1 requires the assumption of the Weibull model in advance, while Figure 1 learns a differential form from data without assuming a model beforehand.

Similar to Figure 3, Figure A2 forecasts the shelf life of myricetin beyond the data used in the fitting process by extrapolating the model coefficients to 250 μM, as represented by the dashed lines in Figure A2a. Despite fitting individual degradation curves well, the Weibull model overestimates the shelf life of myricetin at 250 μM (Figure A2b). Because the Weibull model fits the learning data well as exemplified in Figure A1, it is difficult to anticipate that its forecasting will not be accurate beyond the range of the data used to fit the model. When compared with the results in Figure 3, Figure A2 also suggests that accurately capturing the initiation phase is important for describing myricetin degradation. The initiation phase captured by the differential form Equation (3) identified by Sindy is difficult to observe in the individual degradation curves, but it is critical for accurate long-term predictions.
